# Variations in the 24 h temporal patterns and time budgets of grazing, rumination, and idling behaviors in grazing dairy cows in a New Zealand system

**DOI:** 10.1093/jas/skad038

**Published:** 2023-01-28

**Authors:** Muhammad Wasim Iqbal, Ina Draganova, Patrick Charles Henry Morel, Stephen Todd Morris

**Affiliations:** School of Agriculture and Environment, College of Sciences, Massey University, Private Bag 11–222, Palmerston North 4442, New Zealand; School of Agriculture and Environment, College of Sciences, Massey University, Private Bag 11–222, Palmerston North 4442, New Zealand; School of Agriculture and Environment, College of Sciences, Massey University, Private Bag 11–222, Palmerston North 4442, New Zealand; School of Agriculture and Environment, College of Sciences, Massey University, Private Bag 11–222, Palmerston North 4442, New Zealand

**Keywords:** automated behavior monitoring, behavior patterns, behavior time budgets, grazing dairy cows, individual animal data

## Abstract

This study investigated the variations in the temporal distributions and the lengths of times utilized for grazing, ruminating, and idling behaviors by grazing dairy cows over 24 h. Spring-calved lactating dairy cows (*N* = 54) from three breeds, Holstein-Friesian (HFR), Jersey (JE), and KiwiCross (KC) in different lactations (1st, 2nd, 3rd) and with different breeding worth index values (103 < BW > 151) were selected. The cows were managed through a rotational grazing scheme and milked once a day at 0500 hours. The cows grazed mainly pasture and consumed additional feeds (maize silage and turnips) in the summer and autumn seasons. AfiCollar was used to record grazing and rumination behaviors (min/h) in the individual cows throughout the lactation period (~270 d). The time neither utilized for grazing nor rumination was counted as idling behavior (min/h). A repeat measure design with PROC MIXED was performed in SAS considering the effects of breed, lactation, individual cow, the hour of the day, season, day within the season, and supplementary feed within the season to evaluate the difference in grazing, rumination, and idling behaviors. Hour of the day, season, day within season, and supplementary feed had significant effects on grazing, rumination, and idling behaviors. Regardless of the season and supplementary feed, cows spent most of the daytime grazing and most of the nighttime ruminating. Grazing activity remained consistently high throughout the day with two peaks around dawn and dusk and a short peak around midnight. Rumination activity remained high from the late evening until early morning. Grazing and ruminating patterns were similar between different breeds and lactations, however, JE cows grazed slightly longer than HFR and KC, and first-lactation cows grazed slightly longer than those in higher lactations. The onset and cessation of grazing activity by the cows were adjusted according to varying day lengths by season. Cows finished grazing earlier when they consumed additional supplements or silage along with pasture. Cows from different breed groups and lactations spent most of their 24 h grazing followed by ruminating and idling. Season and supplementary feed potentially affected the variations in behavior time budgets. These findings should support improving measures for grazing management to address pasture allocation and additional feed demands, and animal welfare in varying environmental and/or managemental conditions.

## Teaser

These findings could support the advancements in the measures for pasture and additional feed management to address individual animals’ requirements and could improve animal welfare in varying environmental and managemental conditions

## Introduction

Cows are naturally motivated to carry out certain activities such as grazing and rumination throughout 24 h forming repetitive patterns. These activities are predominant in pasture-based grazing cows and they spend 90% to 95% of their daily time grazing, ruminating, and resting (Kilgour, 2012). Cows do not graze continuously, their crepuscular grazing patterns are distributed in discrete events or “meals” with clear peaks around dawn (after milking) and dusk (Shabi et al., 2005; von Keyserlingk and Weary, 2010). Rumination is the activity performed by cows after grazing, and in grazing cows, its pattern is also circadian but flexible (Gregorini et al., 2006). The collection of grazing or rumination events (also called bouts) within 24 h, therefore, represents the overall time budget specified for that activity per day (Metz, 1975; Gibb, 1998).

In New Zealand, dairy cow production mainly relies on the grazing of pasture, and cows only receive supplementary feeds if required. New Zealand dairy herds are mainly populated with Holstein-Friesian, Jersey, and KiwiCross (Crossbreed of Holstein-Friesian/Jersey) breeds. Climatic conditions and weather in New Zealand are variable and exhibit a four-season pattern. Under these temperate free-range conditions, grazing cows generally exhibit a daily frequency of three to five grazing events (Gibb, 1998). The frequency of grazing fluctuates with the current physiological state of the animal, the grazing method, and the environment (Gregorini, 2012). The choice for a behavioral activity is determined by the current state of the animal and its environment (Mangel and Clark, 1986). Those decisions ascertain how grazing cows invest their time in eating to address their metabolic and nutrient requirements. The factors that affect grazing are believed to influence rumination as well because grazing and rumination are interdependent. Whereas, the potential factors that can alter the rumination pattern are the quality and type of diet and time spent consuming it (Pearce, 1965; Schirmann et al., 2012); as the time budget specified by a cow for ruminating is diet-dependent. The metabolic, diurnal, and seasonal rhythms of grazing and ruminating patterns are influenced by the quality and quantity of pasture, weather, daylight hours, and theoretical food requirements (Corbett, 1953; de Vries and Daleboudt, 1994). Grazing and rumination patterns in pasture grazing cows are also modulated by the addition of supplement feeds and the timing those supplements are offered to the animals (Al-Marashdeh et al., 2018). Furthermore, the variations in grazing and rumination patterns and time budgets could be due to the production level of the animal as well; for instance, compared to low-producing cows, high-producing cows are expected to spend a longer period grazing to fulfill their energy requirements. In addition, variations in grazing and rumination behavior patterns and time budgets might exist in cows with different years of lactation (and production) due to varying energy requirements, and cows of different breeds due to physiological and anatomical differences (Prendiville et al., 2010; Vijayakumar et al., 2017).

How grazing dairy cows distribute their behaviors patterns over 24 h and how the frequency of behavior varies in different cow breeds and lactation groups during different seasons consuming different supplementary feeds is important in understanding their grazing process. Understanding the behavior patterns and time budgets, and their variations is an essential tool to improve the overall grazing management strategies for a pasture-based system. For example, the information can be applied to manage pasture allowance and additional feed resources for day-to-day and even within-a-day operations of the dairy farm throughout the year. Furthermore, the temporal distribution, duration, and intensity of grazing events can also help improve the nutrient supply to grazing cows. The behavior patterns represent benchmarked routines, and the time budgets indicate a net response of cows to their environment. Any prolonged deviations in those patterns or time budgets due to environmental and/or management constraints can result in negative consequences for animal productivity and health (Dittrich et al., 2019). Determining grazing and rumination behavior patterns and time budgets therefore can help to depict a balance in those activities and to evaluate any deviations in those natural behaviors. Also, obtaining this information could further serve as a foundation to develop better management strategies for improving animal productivity and welfare without interfering with animals’ natural behavior.

There is little literature concerning an intriguing question; how to describe the varying behavior patterns in grazing dairy cows and what happens at the individual animal level in the paddock. To the best of our knowledge, there are few studies in the literature to date that have explored the behavior patterns and time budgets in grazing dairy cows (Sheahan et al., 2011; Jochims et al., 2020). Whereas, neither of those studies collected data for long period (e.g., for a complete lactation period), nor discussed the various of factors affecting behavior patterns and time budgets. One of the potential reasons has been the lack of technology to record those behaviors in individual animals continuously for 24 h in a grazing-based system. Most of the research has relied on the use of visual observations to record different behaviors in grazing dairy cows which is a time-consuming procedure. Precision Livestock Farming (PLF) technology offers sensors monitoring the individual animal’s behavior on a real-time basis. Various PLF tools such as collar devices and tags have been developed, validated, and applied to monitor behaviors in grazing cows (Frost et al., 1997; Schirmann et al., 2012; Elischer et al., 2013; Iqbal et al., 2021).

The primary objective of this study was to evaluate the variations in the 24-h behavior patterns and time budgets of grazing, rumination and idling in grazing dairy cows from different breeds and in different lactations, during different seasons, and when fed with different supplements.

## Materials and Methods

### Ethical statement

The study was carried out at Dairy unit 1, Massey University, Palmerston North, New Zealand (Latitude: −41.3009, Longitude: 174.7720). Approval (Protocol No. 18/58) for the care and handling protocols of animals was received from the Animal Ethics Committee, Massey University, New Zealand.

### Grazing area and the local climate

Dairy unit 1 is a pasture-based dairy farm operated through a rotational grazing method with once-a-day milking (0500 hours) and spring calving systems. The farm area consists of 142.7 hectares, further divided into 63 grazing paddocks. The local climate is the temperate type with four seasons. The climatic seasons are classified as spring (September to November), summer (December to February), autumn (March to May), and winter (June to August). The average temperature of the local area was ~14 °C, and rainfall was ~970 mm with 1735 annual sunshine hours and 81% mean relative humidity over the data collection period (NIWA, 2022). The daylight hours in spring, summer, and autumn seasons were 12.5, 14, and 11.5, respectively.

### Grazing animals

Spring calved, pasture grazing lactating dairy cows (*N* = 54) used in the study were a subset of the whole herd (*N* = ~260), they grazed together along with the other cows and altogether managed as one herd. The selection of cows was based on their breed type and lactation number within each breed and breeding worth (BW) index value. BW index value is the measure of the genetic merit of the animal for farm profit (­Gregorini et al., 2013). Eighteen (*N* =18) cows from each of the three breeds, Holstein-Friesian, Jersey, and Holstein-Friesian/Jersey Crossbreed (KiwiCross) were included in the group. The cows included in each breed group were from different lactations (1st, 2nd, and 3rd) with six cows (*N* = 6) from each lactation. The cows within each lactation had varying BW index values (103 < BW > 151). The cows were altogether kept in the same grazing paddocks all the time throughout their lactation period (~270 d) except when brought to the milking shed (~ 2 h per milking) at 0500 hours. All cows from different breeds and in different lactations grazed the same pasture at the same time.

### Feeding the experimental animals

The cows mainly grazed pasture of perennial ryegrass (*Lolium perenne*) mixed with red clover (*Trifolium pretense*) and white clover (*Trifolium repens*). Besides pasture, cows grazed chicory (*Cichorium intybus*) in spring. To meet energy requirements and to cope with the seasonal changes in pasture quality and production (Machado et al., 2005), cows were also fed additional supplements including maize silage (*Zea mays*) and turnips (*Brassica rapa*) on various days during the summer and autumn seasons along with main feed (pasture). Supplementary feeds are used when quality pasture is less available, to fill the feed deficits and to support the cows to maintain energy intake and production (DairyNZ, 2022). The supplements were only used to provide energy when there was insufficient pasture available especially during summer and autumn. Moreover, the purpose of providing supplements to milking cows in autumn is also to achieve calving body condition score (BCS) targets, if the feeds are not supplemented, cows are more prone to lose as quality pasture is insufficient at that time of the year. Maize silage and turnip stems and leaves as such (in situ) were fed around midday in the paddock. The cows had ad libitum access to drinking water.

### Behavior recording

An automated device, AfiCollar (Afimilk Ltd. Kibbutz Afikim, 1514800, Israel) was used in this study to monitor and record the time spent grazing and ruminating by the grazing dairy cows over the lactation period. The device has been validated for measuring grazing and rumination behaviors in grazing dairy cows (Iqbal et al., 2021). The device continuously monitored and recorded the minute-by-minute behavior of the cows for 24 h on a real-time basis. The AfiCollar device had a triaxial (x, y, z) accelerometer-based sensor in a box attached to the collar and positioned on the right side of the animal’s neck. The sensor could identify and classify specific behavior categories such as grazing, and rumination based on the patterns of the animal’s head movements. The data collected by the sensor were analyzed by the collar device using built-in generic algorithms and produced as minute-per-hour (min/h) behavior counts (min/h grazing time and rumination time). The data collected by the AfiCollar device were wirelessly transmitted to a base station through Wi-Fi while cows were in the range of ~500 meters. The data transmission took place once per day when cows were in the shed for milking. The data were downloaded in a Microsoft Excel spreadsheet (Version 2016) from the computer attached to the base station.

### Data collection and preparation

This study collected grazing (min/h) and rumination (min/h) behaviors of grazing dairy cows over the lactation period (~270 d). The lactation period of dairy cows ranged from August to April of the next year (2018–2019) following the typical spring calving system in New Zealand. The AfiCollar device was fitted to the individual cow after the calving event, and it was kept by the cow throughout the lactation period until drying off. The data collection started once the cow calved and ended when the cow was dried off. Minutes per hour spent grazing and rumination by the individual cow were collected for each hour of the day throughout the lactation period, organized, and used as such to perform analysis. The data for the individual animal were separately sorted in Microsoft Excel spreadsheets (Version 2016). The behavior data collected for the individual cow for the individual hour over 24 h were further classified and sorted by breed and lactation of the cow, the season of the year, each day within the season, and supplementary feeding within the season. The overall number of observations registered and used for analysis was 269314.

## Data Analysis

### Hourly behavior patterns

Prior to the analysis, idling behavior was added as a new variable in the data and defined as the time when the animal was neither grazing nor ruminating (Forbes, 1988). Idling behavior expressed as min/h was calculated for the individual cow for each hour by subtracting the time spent grazing and ruminating from 60 (total minutes in an hour).

A repeated measure design using PROC MIXED was performed in SAS (version 9.4, SAS Institute Inc., Cary, NC) fitted with breed × lactation (with three levels each), individual cow within the breed and lactation, the hour of the day, season, the day within the season, and supplementary feeding within the season. The model investigated the differences in grazing (min/h), rumination (min/h), and idling behaviors (min/h) as recently done by another study (Jochims et al., 2020). Grazing, rumination, and idling behaviors were the main dependent variables. Breed and lactation of the cows were the main fixed effects in the model. Individual cows within the breed and lactation were used as a random effect in the model. The day within season and hour of the day were added as repeated measures on the subject cow. Lactation length covered three seasons (spring, summer, and autumn), the season was added in as a fixed factor to test its effect and its interactions with other factors (breed, lactation, hour) on hourly grazing, rumination, and idling behaviors. Cows received different supplementary feeds on different days in the summer and autumn seasons, so, the supplementary feeding within the season was added as a fixed effect in the model. To determine the hourly patterns, the data were summarized and expressed as the least square means of minutes utilized for grazing and rumination within the individual hour (min/h) over 24 h. The least-square means of grazing and rumination behaviors for different breeds, lactations, and seasons for different hours were obtained through breed × hour, lactation × hour, and season × hour interactions respectively in the model. To visualize the grazing and rumination patterns, the least-square means of grazing and rumination for each hour over 24 h were plotted in a line chart using Microsoft Excel (version 2016).

### 24 h behavior time budgets

The outputs by the same model, with the effects of breed, lactation, season, and supplementary feed for the whole ­dataset (without hour category) were used to determine behavior time budgets. The least square means of grazing, rumination, and idling across 24 h were considered as the average time budget for each behavior within 24 h. The means of time budgets for the breed, lactation, season, and supplementary feed within the season for grazing, rumination, and idling behaviors were compared using *P*-values by the same model. The time budget values were summarized and expressed as hours per day as the proportion (%) of the time within 24 h utilized for each behavior.

## Results

### Grazing and rumination hourly patterns

**Figure 1.**
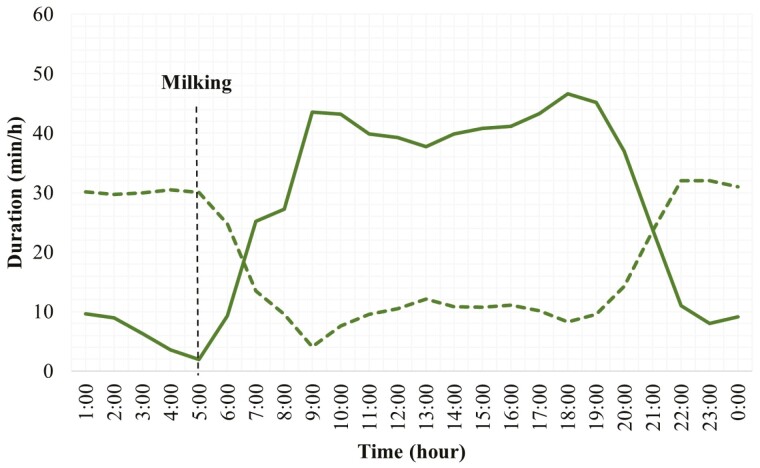
Duration of grazing and rumination behaviors for each hour over 24 h in grazing dairy cows (*N* = 54). (Compact line shows grazing activity and the dotted line shows rumination activity).

Hour of the day had a significant effect on the hourly grazing and rumination patterns of grazing dairy cows (Table 1). The 24-h temporal distributions of grazing and rumination activities over 24 h and significant differences among different hours are shown in Figure 1 and Table S1. Regardless of the season, supplementary feed type, breed type, and lactation number, grazing dairy cows spent most of the daytime grazing and most of the nighttime ruminating. Grazing activity started early in the morning, gradually increased, and remained high throughout the day with a decline in the evening. The intensity of grazing (The minutes spent grazing within an hour) was higher especially in the morning and in the late afternoon until evening. The evening grazing peak was slightly higher than that in the morning. Grazing activity remained low over the night hours except a short peak around midnight. In contrast to grazing, rumination activity in grazing dairy cows gradually increased from the late evening and consistently remained high until the early morning when cows were brought to the milking shed (Figure 1). Rumination activity remained low throughout the daytime.

**Table 1.** *P*-values for the effects of breed, lactation, hour, season, day within season feed within the season, and their interactions on grazing (min/h) and rumination (min/h), and idling (min/h) behaviors using a mixed effects model with the cow (*N* = 54) as a random factor

Effect	*P*-value		
	Grazing, min/h	Rumination, min/h	Idling, min/h
Breed	0.0638	0.3544	0.6672
Lactation	0.8161	0.5115	0.3645
Breed*lactation	0.0785	0.9216	0.5352
Hour	<0.0001	<0.0001	<0.0001
Season	<0.0001	<0.0001	<0.0001
Day (Season)	<0.0001	<0.0001	<0.0001
Feed (Season)	0.0016	0.0246	0.6781
Breed*season	<0.0001	<0.0001	<0.0001
Lactation*season	<0.0001	<0.0001	<0.0001
Breed*hour	<0.0001	<0.0001	<0.0001
Lactation*hour	<0.0001	<0.0001	<0.0001
Season*hour	<0.0001	<0.0001	<0.0001
Feed (Season)*hour	<0.0001	<0.0001	<0.0001
Breed*lactation*season	<0.0001	<0.0001	<0.0001
Breed*lactation*season*hour	<0.0001	<0.0001	<0.0001

(The significance of the *P*-value was set at 0.05. *Indicates interaction. R-square (R^2^) values of the models for grazing, rumination, and idling were 0.55, 0.43, 0.33, respectively).

**Figure 2.**
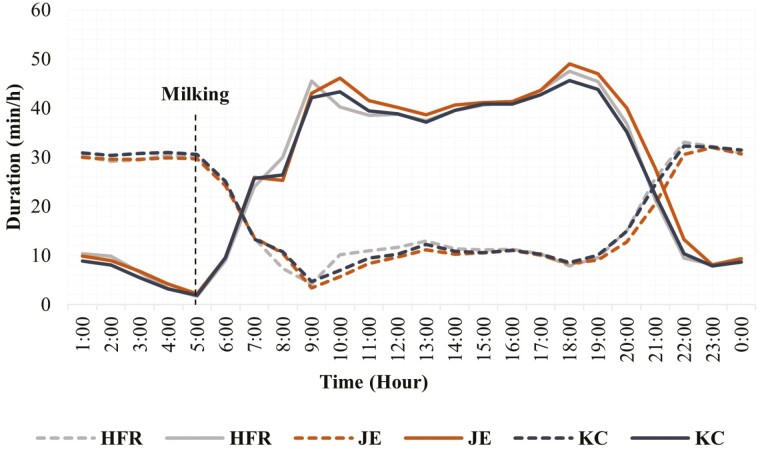
Duration of grazing and rumination behaviors for each hour over 24 h in different breeds of grazing dairy cows (*N* = 54). (Compact lines show grazing activity and the dotted lines show rumination activity. HFR = Holstein-Friesian, JE = Jersey, and KC = KiwiCross).

The breed effect was not significant for both grazing and rumination; however, the breed had a statistical interaction with the hour of the day (Table 1). The hourly grazing and rumination patterns for different breeds based on the least square means of breed × hour interactions are presented in Figure 2 and Table S2. The temporal pattern of grazing differed among breeds mainly around midnight, morning, and evening (e.g., 2200, 0200, 0400, 2000 hours.) and remained similar for most of the day hours. The temporal pattern of rumination differed among breeds mainly around midday (e.g., 1100 hours) and dawn (e.g., 0400 hours) and remained similar for most of the night hours. Jersey (JE) cows showed a longer grazing period with more consistent activity compared to Holstein-Friesian (HFR) and KiwiCross (KC) cows. JE cows’ morning and evening grazing peaks were one hour later than that of HFR cows. HFR cows showed slightly elevated rumination activity after the morning grazing peak.

**Figure 3.**
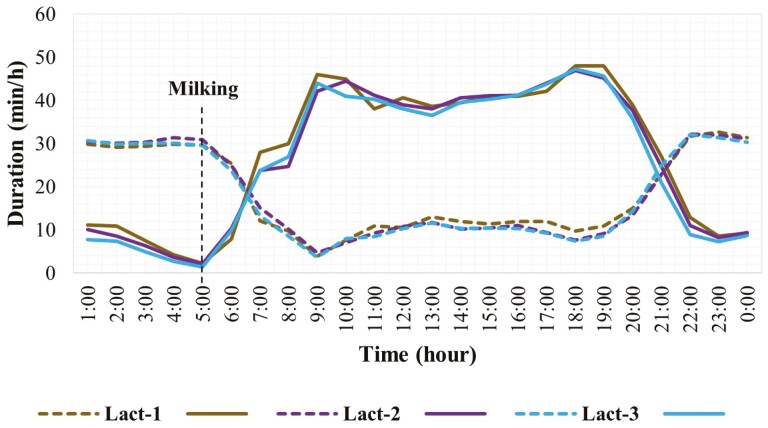
Duration of grazing and rumination behaviors for each hour over 24 h in grazing dairy cows (*N* = 54) in different lactations. (Compact lines show grazing activity and the dotted lines show rumination activity. Lact-1, Lact-2, and Lact-3 represent first, second, and third lactation cows, respectively).

The lactation effect was not significant for both grazing and rumination, however, lactation had a statistical interaction with the hour of the day (Table 1). The hourly grazing and rumination patterns for different lactations based on the least square mean values (lactation × hour) are presented in Figure 3 and Table S2. The temporal pattern of grazing differed among cows in different lactations mainly around midnight, morning, midday, and evening hours (e.g., 0100, 0600, 1300, 2100 hours.) and remained similar for most of the day hours. The temporal pattern of rumination differed among lactations mainly around the evening (e.g., 1700, 1800 hours.) and remained similar for most of the day and night hours. The first lactation cows showed more intense and longer grazing activity compared to cows in 2nd or 3rd lactation. Breed and lactation of cows did not show a statistical interaction with each other for hourly grazing and rumination pattern.

**Figure 4.**
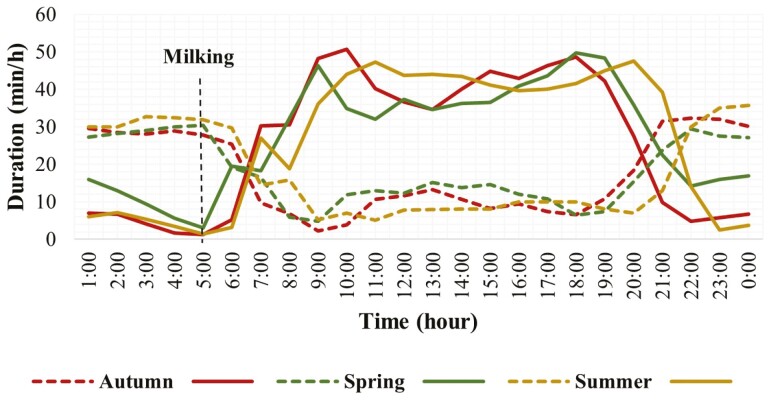
Duration of grazing and rumination behaviors for each hour over 24-h in grazing dairy cows (*N* = 54) for different seasons during the experimental period. (Compact lines show grazing activity and the dotted lines show rumination activity).

Season effect was significant for both grazing and rumination, and season had a statistical interaction with the hour of the day (Table 1). The hourly grazing and rumination patterns for different seasons based on the least square mean values (season × hour) are presented in Figure 4 and Table S3. The temporal distribution for grazing and rumination activities varied among seasons for most of the hours over 24 h (Table S3). The behavior patterns varied according to the seasonally varying day lengths or daylight hours. Grazing activity in dairy cows started and finished earlier in autumn, whereas it finished later in spring and summer with a 2-hour difference. Grazing activity was more consistent with an early morning steep line and intense peak in autumn. Rumination activity during the daytime was slightly higher in spring compared to autumn and summer.

**Figure 5.**
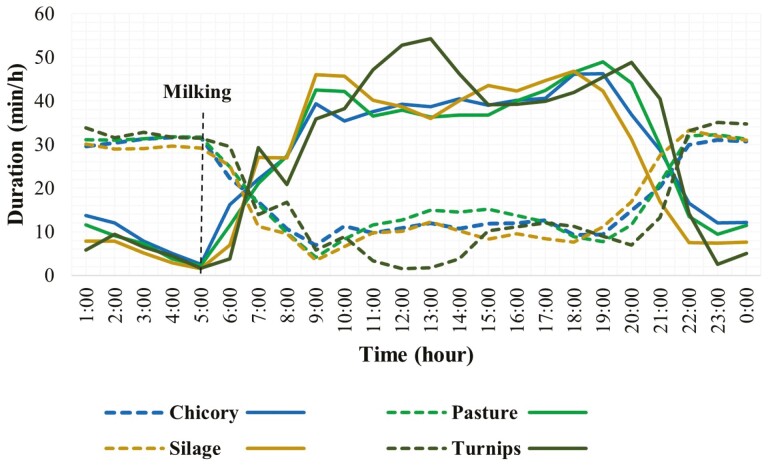
Duration of grazing and rumination behaviors for each hour over 24 h in grazing dairy cows (*N* = 54) consuming different feeds. (Compact lines show grazing activity and the dotted lines show rumination activity).

Supplementary feed had a significant effect on both grazing and rumination, and feed had a statistical interaction with the hour of the day (Table 1). The hourly grazing and rumination patterns for different supplementary feeds based on the least square mean values (feed × hour) are presented in Figure 5 and Table S4. Although feed had a significant effect, the temporal patterns of grazing and rumination behaviors over 24 h not much varied with varying supplementary feeds except for turnips (Figure 5). Grazing was more consistent with less resting interval when cows grazed on pasture or chicory compared to when they consumed turnips, silage, and supplements as additional feeds. Among additional feeds, when consumed turnips, the cows showed more intense, continuous, and longer grazing activity until late in the evening. Whereas cows always finished grazing earlier in the evening when they consumed supplements or silage as additional feeds. Rumination activity was higher during daylight hours when cows grazed solely pasture or chicory compared to that for other feed types.

### Grazing, rumination, and idling time budgets

**Figure 6.**
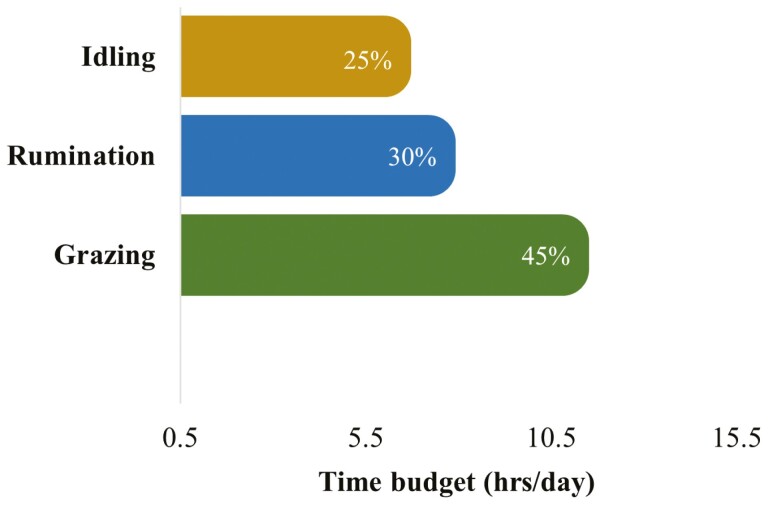
The overall 24-h (per day) time budgets for grazing, rumination, and idling behaviors in grazing dairy cows (*N* = 54). (This figure is based on the least square means for grazing, rumination, and idling behaviors over 24 h across the whole lactation period. The values within the bars indicate the proportion (%) of time utilized for each behavior within 24 h).

**Figure 7.**
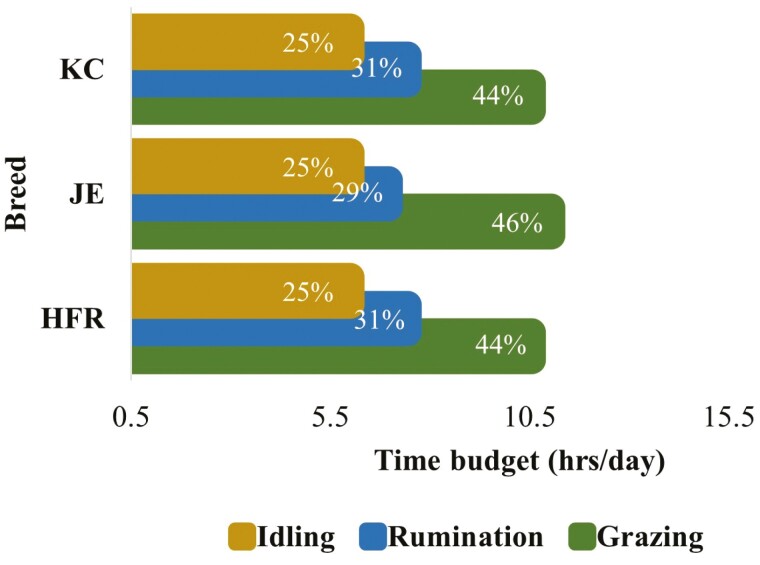
The 24-h (per day) time budgets for grazing, rumination, and idling behaviors for grazing dairy cow (*N* = 54) of different breeds. (HFR = Holstein-Friesian, JE = Jersey, KC = KiwiCross. This figure is based on the least square means for grazing, rumination, and idling behaviors over 24 h across the whole lactation period. The values within the bars indicate the proportion (%) of time utilized for each behavior within 24 h).

**Figure 8.**
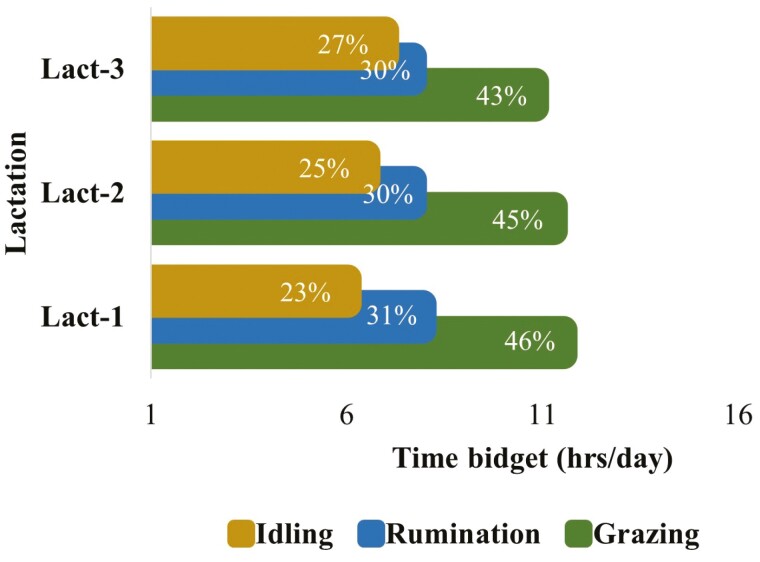
The 24-h (per day) time budgets for grazing, ruminating, and idling behaviors for grazing dairy cows (*N* = 54) in different lactations. (Lact-1, Lact-2, and Lact-3 represent cows in first, second, and third lactations, respectively. This figure is based on the least square means for grazing, rumination, and idling behaviors over 24 h across the whole lactation period. The values within the bars indicate the proportion (%) of time utilized for each behavior within 24 h).

Grazing, rumination, and idling time budgets for 24 h mainly varied due to the effects of season and feed within the season, however, the effects of breed and lactation remind nonsignificant (Table 1). Irrespective of breed, lactation, season, and supplementary feed, cows spent most of their time grazing followed by ruminating and idling within 24 h (Figure 6). The 24 h time budgets for grazing, rumination, and idling were similar between Holstein-Friesian (HFR), Jersey (JE), and KiwiCross (KC) cows (Figure 7). The 24 h time budgets for grazing, rumination, and idling between cows in different lactations were similar (Figure 8). However, there was a slight increase in the idling time budget and a decrease in the grazing time budget in the cows with consecutive higher lactation.

**Figure 9.**
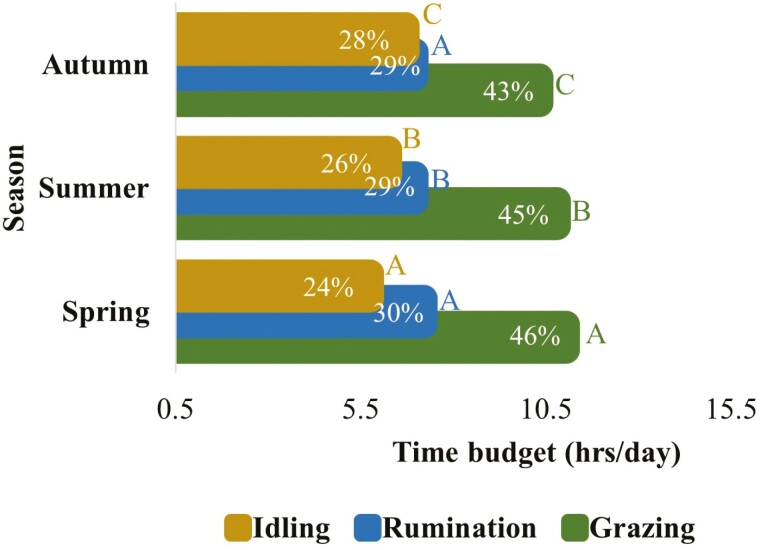
The 24-h (per day) time budgets for grazing, rumination, and idling behaviors in grazing dairy cows (*N* = 54) in different seasons over the lactation period. (Means that do not share a common letter are signiﬁcantly different for the signiﬁcance level set at the *P*-value of 0.05. This figure is based on the least square means for grazing, rumination, and idling behaviors over 24 h across the whole lactation period. The values within the bars indicate the proportion (%) of time utilized for each behavior within 24 h).

**Figure 10.**
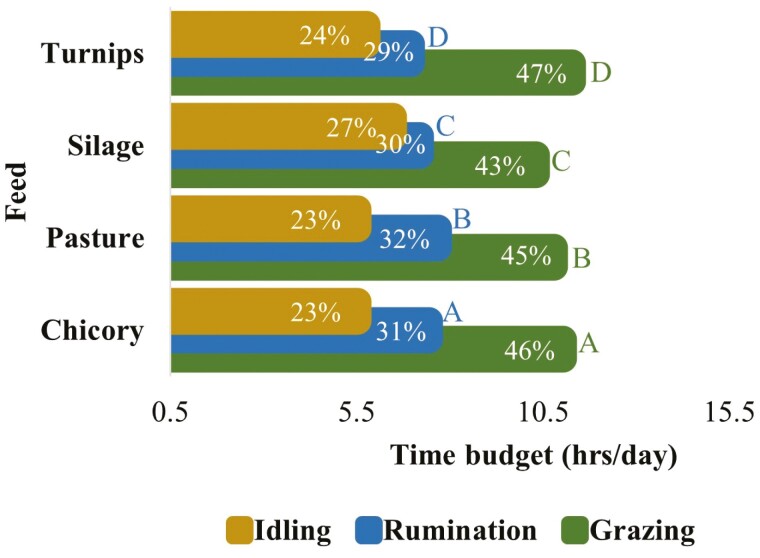
The 24-h (per day) time budgets for grazing, rumination, and idling behaviors in grazing dairy cows (*N* = 54) when fed with different supplementary feeds. (Means that do not share a common letter are signiﬁcantly different for the signiﬁcance level set at the *P*-value of 0.05. This figure is based on the least square means for grazing, rumination, and idling behaviors over 24 h across the whole lactation period. The values within the bars indicate the proportion (%) of time utilized for each behavior within 24 h).

The 24 h time budgets of grazing dairy cows for grazing, rumination, and idling significantly varied during different seasons (Figure 9). Cows spent most of their time grazing followed by ruminating in all seasons, however grazing and ruminating time budgets declined from spring towards autumn. The idling time budget increased from spring to autumn, with being highest in autumn and lowest in spring. The 24 h time budgets for grazing and ruminating behaviors significantly varied when cows consumed supplement feeds in addition to pasture (Figure 10). However, supplementary feed did not affect idling time budgets. The grazing time budgets were similar for pasture and chicory. However, the grazing time budget was relatively higher when cows received turnips. The rumination time budget did not much vary for different feeds. However, the idling time budget was comparatively lower when cows received turnips and higher when they consumed silage.

## Discussion

This study examined the trends and variations in the temporal patterns of grazing and rumination, and idling behaviors and their time budgets over 24 h in grazing dairy cows. The study was unique as it used a large behavior data set collected longitudinally over the whole lactation period. Grazing and rumination behaviors were continuously measured using an automated collar device (AfiCollar). All cows of different breeds and in lactations were kept as one herd in the same paddock and grazed the same pasture; therefore, the herding instinct of cattle may have influenced the differences in behaviors among breeds and lactation. Similarly, season and supplementary feeding were linked with each other in a way that supplementary feed was confounded within the season. Therefore, the confounding may have partly influenced the effects of season and supplementary feeds.

Results indicated that regardless of breed type, lactation number, season, and supplementary feeds, pasture-grazing dairy cows spent most of the daylight hours grazing and most of the night hours ruminating; there was a short-lived grazing peak around midnight. The grazing intensity was highest in the morning after milking and, in the evening, before the start of the dark period. Previous studies have reported similar findings where most of the grazing activity occurred immediately following the milking and remained high throughout the day with a smaller peak around midnight (Hughes and Reid, 1951; Orr et al., 2001; Gibb et al., 2002). The higher intensity of grazing in the morning and evening in the current study was consistent with the previous reports. Dairy cows in New Zealand tend to exhibit a cyclic pattern and the temporal distribution of grazing and rumination periods largely depends upon milking time and the time of sunrise and sunset (Brumby, 1959; Krysl and Hess, 1993). When the sun is close to the horizon during sunrise and sunset periods, the different ratio of shorter and longer wavelengths compared to that at midday has been suggested to have a stimulatory effect on appetite (Linnane et al., 2001; Gregorini et al., 2006). In addition, the natural evacuation of digesta from the gut leads to the smallest ruminal pool size early in the morning and could potentially generate hunger; this explains the high motivation to graze at sunrise. The dusk grazing event in the current study was slightly more intense than that of the dawn. Previous studies have already noted that the dusk grazing event is longer and more intense (Orr et al., 1997; Gibb, 1998). Cows spent 25% and 40% of their daily grazing time during dawn and dusk respectively with a higher bite rate and bite mass for the latter (Taweel et al., 2004). Moreover, the physical characteristics of pasture and environmental conditions may also contribute to this grazing pattern (Jochims et al., 2020). Furthermore, the intense desire for grazing by the cows in the evening is to maximize intake, when herbage is at its peak nutritive value, and to provide optimum feed for digestion during the night (Taweel et al., 2004). Thus, diurnal changes in herbage quality might also be playing a role in driving the intense and extended grazing event at the dusk. In addition, studies had already suggested that grazing activity predominantly occurs during the daytime because of thermal comfort and the photoperiod effect (Hogan et al., 1987; Gibb et al., 1998; Linnane et al., 2001). In a previous study (Phillips and Schofield, 1989), increased numbers of feeding bouts were found when cows were provided extra light during short days. Another study noted a disruption in the grazing pattern of dairy cows during a total solar eclipse (Rutter et al., 2002). Thus, the absence or presence of light significantly affects grazing activity in grazing dairy cows. Findings of a study by (Champion et al., 1994) suggested that warmer environmental temperature is the reason most of the grazing activity occurred during the daytime, as the temperature is relatively colder at night. However, grazing events over the night, as observed in the current study, are also essential for the cows to retain their metabolic heat production through rumen fermentation (Forbes, 2007). The shorter and less intense grazing event occurring at night contributes about 10% to 15% to the daily grazing time and approximately 10% to the daily herbage intake (Stobbs, 1977; Krysl and Hess, 1993).

Rumination is a natural behavior of cattle and the most important activity in the ruminants after grazing (Realini et al., 1999). The distribution of rumination time by grazing dairy cows over 24 h in the current study showed that most rumination occurred at nighttime. However, a few short-lived rumination events during the daytime were also found. These findings were supported by previous studies (Newman et al., 1995; Prins, 1996; Houtman and Dill, 1998). Cows performed most of the rumination activity at night while resting (Albright, 1993). A few other studies also reported that rumination activity in grazing dairy cows predominantly occurred at night but it was recorded both during day and night (Ternman et al. 2018, 2019). Rumination tends to follow a daily pattern and cows spend a larger proportion of time ruminating at night and after intense feeding. Moreover, rumination is more likely to occur when cows are lying down, making it important to ensure that dairy cows have adequate, comfortable space.

Holstein-Friesian, Jersey, and KiwiCross breeds showed a similar distribution of grazing and ruminating patterns over 24 h. It has been reported that when grazing, cattle often synchronize their behavior in a way that animals as a group feed, ruminate, and rest at the same time (Miller and Wood-Gush, 1991). The synchronization of behavior has also been observed in indoor-housed cows (Curtis and Houpt, 1983). Although grazing patterns were similar between breeds, JE cows showed slightly longer grazing activity compared to HFR and KC cows. Studies suggest that the eating and rumination patterns of Jerseys are different from those of larger dairy breeds (HFR) in a manner consistent with greater intake capacity and rate of digesta passage (Welch et al., 1970; Aikman et al., 2008). Also, grazing frequency and bite mass are influenced by the constraints due to the anatomy of the animal including mouth and body size (Rook, 1999). Jersey cows have short stature with a small body and mouth size; therefore, JE cows’ bite mass is smaller. Due to smaller bite mass JE cows take a longer time to fulfill their feed demands (Dürst et al., 1993; Rook, 1999). The current study found a short-term rumination period after an intensive morning grazing peak in HFR cows; this has been previously reported (Sheahan et al., 2011). A possible explanation for this could be the release of neuroendocrine factors secreted in response to the presence of food in the digestive tract (Faverdin, 1999), which is implicated as a satiety factor. The release of several neuroendocrine proteins associated with a hunger or satiety role coincides with the beginning or cessation of a meal (Roche et al., 2008).

The overall temporal patterns of grazing and rumination over 24 h were similar between grazing dairy cows in different lactations. However, the continuous and more intense grazing activity during daylight hours by first lactation cows can be justified as they have a smaller body size, take smaller bites, and eat slower, hence graze longer compared to mature cows to address their nutritional demands. Furthermore, first-lactation cows are still in a growing phase and need additional energy to support growth in addition to energy for maintenance requirements (Grant and Albright, 2001).

The current study found variations in the onset and cessation of grazing activity for varying day lengths during different seasons. Grazing activity occurred mainly in daylight during different times of the year, but the day length affected changes in the grazing patterns. This might be a consequence of the interaction of photoperiod and environmental temperatures. Seasonal variations in photoperiod affect grazing behavior and resultant herbage intake (Rhind et al., 2002). Compared to longer days in spring and summer, cows finished their grazing activity earlier during shorter days in autumn with relatively more intense grazing peaks. This can be justified as cows had the higher motivation to fill their rumen before the start of nightfall. Cows also adjusted the intensity of their grazing activity according to the day length; it was more vigorous during the short days in autumn than that on long days in spring or summer. The backward shift of grazing peaks in autumn was also partly due to the absence of daylight savings that was observed during spring and summer. Another study (Sheahan et al., 2011) also reported that animals adapted their grazing habit according to day length and started early during short days so, they have more grass intake to address their satiety needs. An adaptation based on the variation in day length could be an increase in the duration of grazing events and a decrease in the number of meals during short days or an increase in the number of mealtimes at night (Gregorini et al., 2006); However, the duration of the grazing event remains constant regardless of the actual time of sunset (Rutter et al., 2002). Moreover, the different grazing peaks during different seasons demonstrate the ability of animals to adapt their ingestive activity in daylight, reserving most rumination and rest activities for periods of darkness to maintain their welfare. It is also due to adaptation to avoid predation and to cope with reduced eyesight during the night. Furthermore, the midnight grazing peak was slightly longer during spring. The temperature distribution over 24 h is different especially in different seasons. spring season is cooler with an average temperature of 19 °C compared to summer (25 °C) and autumn (21 °C). Grazing events over the night are also necessary for the animals to maintain their metabolic heat production (by rumen fermentation) during cool seasons (Forbes, 2007). Therefore, to maintain their welfare, animals distributed their grazing activity differently overnight during different seasons. Rumination activity after the morning peak in spring was higher compared to that in summer. There is a natural drive for rumination after intense grazing, therefore, the cows were taking rest (with a pause in grazing) after the morning grazing intense peak and ruminating a little while resting. Also, after the first meal (morning grazing peak), animals decrease the time spent grazing, probably because of rumen filling (Demment et al., 1995). This was consistent with a recent study where animals showed higher rumination activity during day in spring compared to that in summer (Jochims et al., 2020).

The cows mainly grazed pasture; however, different supplements were fed to the cows in different seasons at different times of day to address the feed demands. Time of day and feed type are confounded and their effects on each other cannot be separated. Therefore, the time of day when the supplement is fed would likely have the greater impact on grazing behavior rather than the supplement itself in the current study. Variations in the temporal distributions of grazing and rumination patterns were also linked with the different supplementary feeds consumed by animals during different seasons. The variations in grazing and rumination activities were high when animals consumed supplements along with pasture. Supplement and silage were usually fed to cows in summer as additional feeds to fulfill their energy demands as pasture quality was low at that time. Although the days were longer in summer, the cows finished their grazing activity earlier in the evening and started ruminating. It has already been reported that quantity, quality, and the timing of feeding supplements greatly influence behavior patterns of pasture-grazing cows (Heublein et al., 2017). It has been suggested that the role of dawn grazing preference is certainly active in shaping the daily grazing pattern (Gregorini et al., 2006). As silage and supplements were fed to the cows at dawn after milking that might have supported the early fulfillment of nutritional requirements and motivated the early cessation of grazing activity. Cows received turnips during mid-day which resulted in the higher intensity of grazing in the afternoon as timing and delivery of fresh feed are highly stimulating factors for cows to perform grazing (DeVries and von Keyserlingk, 2005).

Time budgets for grazing, rumination, and idling activities over 24 h were quite similar between cows from different breed groups and cows in different lactations. However, the proportion of time spent chewing (grazing plus rumination) over 24 h decreased and the amount of time spent idling increased in the cows with consecutive higher lactation numbers. A recent report that cows in higher lactations spend more time lying compared to cows in early lactations (Munksgaard et al., 2020). Another study also reported the effect of parity on-time budgets with a higher proportion of time spent idling in higher lactation cows compared to lower lactation cows (Sepúlveda-Varas et al., 2014). Thus, the findings of the current study agree with those reported by the previous studies.

Seasonal variations in 24 h time budgets in grazing dairy cows were prominent. The percentage of time spent chewing increased from spring (when animals calved, early lactation) reached the peak at the end of spring and started declining in summer. The chewing time budget was further down and ­lowest in autumn when animals were ready to be dried off and received supplement feeds; whereas the proportion of time spent idling was just the opposite of chewing. Variations in behavior time budgets were apparent in the autumn season when cows were about to be dried off, and during the time cows received supplements; this resulted in a higher amount of time spent idling. The amount of time spent idling was highest in autumn when most of the animals were about to be dried off and had less feed requirements, and the cows received supplement feeds as well. A recent study (Maselyne et al., 2017) reported a similar finding for lying time that is consistent with the results of this study. Both grazing and ruminating time budgets had higher values in spring and summer at the start and peak of the lactation period and reached a minimum level in autumn when animals were going to be dried off. Two previous studies have recently reported the same pattern in eating and ruminating time budgets in dairy cows (DeVries et al., 2003; Norring et al., 2014). Few other studies have also found a comparatively longer proportion of time spent grazing during the initial weeks of lactation in spring than that in summer or autumn (DeVries et al., 2003; Munksgaard et al., 2020). It has been reported that both milk yield and herbage intake (indicated by grazing time budget) increase during the first few weeks of lactation, and gradually decrease towards the end of lactation (Bossen et al., 2009). Furthermore, both herbage intake and time spent grazing in dairy cows increase during the early lactation and decline towards the end of lactation, going parallel with the milk production curve (Kertz et al., 1991). The slight reduction in the proportion of time spent grazing in summer could be due to the high-temperature humidity index that could have induced heat stress and resulted in reduced grazing time (Kidane et al., 2018).

The time budgets for grazing and rumination did not vary when animals were fed on pasture, chicory, or silage, although, idling time was higher when animals consumed chicory, silage, and supplements. In a previous report, compared with perennial ryegrass, as the dietary proportion of chicory increased, cows spent more time idling and less time ruminating and increased ingestive mastications five and three times for chicory, respectively (Gregorini, 2012). Variation in time budget was quite obvious when cows were fed on supplements. The lowest proportion of time spent chewing when cows received supplements was because their nutritional requirements were addressed by the energetic supplements so, they spent less time grazing and subsequently less time ruminating. Therefore, the proportion of time spent idling was highest when cows received supplements. Similar findings have already been reported (Sheahan et al., 2011; Hills et al., 2015).

## Conclusion

Grazing dairy cows spend most of the daytime grazing to maximize energy intake and most of the nighttime ruminating the grazed herbage. Grazing activity appears to be high at dawn and at dusk with the latter being more intense. Grazing and ruminating patterns are not different between different breed groups although, Jersey cows spend a relatively long time grazing and Holstein-Friesian cows spend a longer time ruminating. Cows in the first lactation graze continuously with fewer resting intervals compared with those in higher lactations. Time of the day and seasonal variations suggested that grazing cows can adjust their behavior patterns according to day lengths. The diurnal changes in herbage quality might also be playing a role in driving the behavior patterns that need further exploration. Cows spend less time grazing and ultimately less time ruminating when pasture has been supplemented with additional feeds. Most of the time over 24 h is spent grazing followed by ruminating and idling in dairy cows in a pasture-based system. Time budgets for the behavior activities over 24 h do not vary much between breed or lactation groups. Whereas variations in time budgets are observed during different seasons and for different supplementary feeds. The analysis of daily behavior patterns can be helpful in support to predict and addressing the behavioral needs of different animals in different climatic conditions by molding the management practices according to the daily behavioral needs. Thus, using the temporal distribution in management strategies to drive the 24 h supply of nutrients can be a potential tool in grazing management.

## Supplementary Material

skad038_suppl_Supplementary_Tables

## Abbreviations

HFR: Holstein-Friesian
JE: Jersey
KC: KiwiCross
