# Supplementary material for: Variations in the 24 h temporal patterns and time budgets of grazing, rumination, and idling behaviors in grazing dairy cows in a New Zealand system
Source: J Anim Sci. 2023 Jan 28;101:skad038. doi: 10.1093/jas/skad038 (PMC9994596; doi:10.1093/jas/skad038)
Supplement: skad038_suppl_Supplementary_Tables [file skad038_suppl_supplementary_tables.docx]

**Table S1.** Least square means ± Standard errors of means for grazing (min/h) and rumination (min/h) for each hour over 24 hours period.

| **Hour** | **Grazing (min/h)** | **Rumination (min/h)** |
| --- | --- | --- |
| 1:00 | 16.9±0.10^K^ | 29.3±0.11^CD^ |
| 2:00 | 16.3±0.09^LM^ | 28.8±0.11^D^ |
| 3:00 | 14.7±0.08^N^ | 29.2±0.11^CD^ |
| 4:00 | 13.1±0.06^O^ | 29.6±0.10^BC^ |
| 5:00 | 12.1±0.04^P^ | 29.4±0.10^BCD^ |
| 6:00 | 15.9±0.08^K^ | 24.1±0.11^E^ |
| 7:00 | 24.5±0.12^I^ | 15.5±0.09^G^ |
| 8:00 | 26.8±0.13^G^ | 12.9±0.08^JK^ |
| 9:00 | 40.8±0.14^B^ | 9.1±0.06^N^ |
| 10:00 | 39.9±0.16^C^ | 11.8±0.09^M^ |
| 11:00 | 37.7±0.17^E^ | 12.8±0.10^KL^ |
| 12:00 | 38.2±0.16^DE^ | 13.2±0.10^JK^ |
| 13:00 | 36.8±0.16^F^ | 14.5±0.11^H^ |
| 14:00 | 38.2±0.15^DE^ | 13.6±0.10^I^ |
| 15:00 | 38.5±0.15^D^ | 13.8±0.10^I^ |
| 16:00 | 39.1±0.15^C^ | 13.7±0.10^I^ |
| 17:00 | 40.7±0.15^B^ | 13.2±0.10^IJ^ |
| 18:00 | 43.8±0.14^A^ | 11.5±0.09^M^ |
| 19:00 | 43.4±0.13^A^ | 11.9±0.09^L^ |
| 20:00 | 37.4±0.17^F^ | 15.6±0.12^G^ |
| 21:00 | 27.5±0.15^H^ | 22.4±0.13^F^ |
| 22:00 | 17.6±0.10^J^ | 30.4±0.12^A^ |
| 23:00 | 16.1±0.09^M^ | 30.5±0.11^A^ |
| 0:00 | 16.6±0.10^KL^ | 29.9±0.11^B^ |

Note. This table is linked to Figure 1. The means that do not share a common letter are signiﬁcantly different for the signiﬁcance level set at the p-value of 0.05.

**Table S2.** Least square means ± Standard errors of means of grazing (min/h) and rumination (min/h) for each hour over 24 hours period for different breeds and lactation numbers.

|  | **Grazing (min/h)** | | | **Rumination (min/h)** | | | **Grazing (min/h)** | | | **Rumination (min/h)** | | |
| --- | --- | --- | --- | --- | --- | --- | --- | --- | --- | --- | --- | --- |
| **Hour** | **HFR** | **JE** | **KC** | **HFR** | **JE** | **KC** | **Lact-1** | **Lact-2** | **Lact-3** | **Lact-1** | **Lact-2** | **Lact-3** |
| 1:00 | 16.5±0.49^A^ | 16.6±0.50^A^ | 15.6±0.36^A^ | 29.0±0.66^A^ | 28.7±0.67^A^ | 29.7±0.48^A^ | 17.2±0.42^A^ | 16.4±0.46A | 15.1±0.49^B^ | 28.5±0.56^A^ | 29.5±0.61^A^ | 29.5±0.66^A^ |
| 2:00 | 16.8±0.48^A^ | 16.5±0.49^A^ | 15.7±0.35^B^ | 27.5±0.69^A^ | 28.2±0.70^A^ | 29.1±0.50^A^ | 17.5±0.41^A^ | 16.0±0.44^B^ | 15.5±0.48^B^ | 27.8±0.59^A^ | 28.8±0.63^A^ | 28.2±0.69^A^ |
| 3:00 | 15.0±0.43^A^ | 15.3±0.43^A^ | 14.2±0.31^B^ | 28.3±0.69^A^ | 28.0±0.70^A^ | 29.4±0.49^A^ | 15.5±0.36^A^ | 14.9±0.39^A^ | 14.2±0.43^B^ | 27.9±0.58^A^ | 28.9±0.63^A^ | 28.7±0.69^A^ |
| 4:00 | 13.2±0.23^A^ | 13.6±0.24^A^ | 12.9±0.17^B^ | 29.1±0.69^A^ | 28.5±0.71^A^ | 29.8±0.51^B^ | 13.4±0.19^A^ | 13.3±0.22^A^ | 13.1±0.24^A^ | 28.7±0.59^A^ | 30.1±0.64^A^ | 28.6±0.69^A^ |
| 5:00 | 12.2±0.15^A^ | 12.3±0.14^A^ | 12.1±0.10^A^ | 28.5±0.78^A^ | 28.3±0.79^A^ | 29.5±0.56^A^ | 12.2±0.12^A^ | 12.3±0.13^A^ | 12.2±0.14^A^ | 28.6±0.66^A^ | 29.5±0.71^A^ | 28.1±0.77^A^ |
| 6:00 | 15.8±0.21^A^ | 16.1±0.21^A^ | 15.9±0.15^A^ | 24.2±0.78^A^ | 23.2±0.79^A^ | 24.5±0.56^A^ | 15.0±0.17^A^ | 16.2±0.19^B^ | 16.6±0.21^B^ | 24.4±0.66^A^ | 24.5±0.71^A^ | 22.9±0.78^A^ |
| 7:00 | 24.4±0.63^A^ | 26.1±0.64^A^ | 26.0±0.46^A^ | 15.20.78^A^ | 14.3±0.79^A^ | 14.8±0.56^A^ | 27.5±0.53^A^ | 24.4±0.58^A^ | 24.6±0.63^A^ | 14.0±0.66^A^ | 16.1±0.72^A^ | 14.2±0.78^A^ |
| 8:00 | 28.9±1.64^A^ | 25.9±1.68^A^ | 27.0±1.18^A^ | 11.3±0.87^A^ | 12.5±0.89^A^ | 13.5±0.63^A^ | 29.9±1.39^A^ | 25.5±1.51^B^ | 26.4±1.63^B^ | 12.5±0.74^A^ | 13.1±0.80^A^ | 11.7±0.87^A^ |
| 9:00 | 41.5±1.05^A^ | 40.4±1.07^A^ | 40.4±0.76^A^ | 9.4±0.37^A^ | 8.7±0.38^A^ | 9.61±0.27^A^ | 41.9±0.89^A^ | 39.3±0.96^A^ | 41.2±1.05^A^ | 9.0±0.31^A^ | 9.6±0.34^A^ | 9.0±0.37^A^ |
| 10:00 | 37.9±1.14^A^ | 42.1±1.16^B^ | 40.8±0.82^A^ | 13.5±0.67^A^ | 10.5±0.68^A^ | 11.2±0.49^A^ | 40.4±0.97^A^ | 41.2±1.04^A^ | 39.3±1.14^A^ | 12.0±0.57^A^ | 11.4±0.62^A^ | 11.8±0.68^A^ |
| 11:00 | 36.1±0.94^A^ | 38.3±0.96^A^ | 37.3±0.68^A^ | 14.0±0.58^A^ | 11.9±0.59^B^ | 12.9±0.41^A^ | 35.8±0.79^A^ | 38.6±0.86^A^ | 37.3±0.94^A^ | 14.1±0.49^A^ | 12.7±0.53^A^ | 12.1±0.58^B^ |
| 12:00 | 38.7±0.77^A^ | 39.3±0.79^A^ | 39.2±0.56^A^ | 13.3±0.58^A^ | 12.0±0.59^A^ | 12.2±0.42^A^ | 39.9±0.66^A^ | 39.6±0.71^A^ | 37.7±0.77^A^ | 12.6±0.49^A^ | 12.4±0.53^A^ | 12.5±0.58^A^ |
| 13:00 | 36.5±0.71^A^ | 37.3±0.73^A^ | 36.5±0.52^A^ | 14.7±0.59^A^ | 13.4±0.61^A^ | 14.3±0.43^A^ | 37.5±0.60^A^ | 37.6±0.66^A^ | 35.2±0.72^B^ | 14.6±0.51^A^ | 13.7±0.55^A^ | 13.9±0.59^A^ |
| 14:00 | 37.5±0.68^A^ | 38.0±0.69^A^ | 37.8±0.49^A^ | 13.6±0.53^A^ | 12.9±0.54^A^ | 13.3±0.38^A^ | 37.5±0.58^A^ | 38.6±0.62^A^ | 37.3±0.68^A^ | 14.1±0.45^A^ | 12.9±0.48^A^ | 12.9±0.53^A^ |
| 15:00 | 39.4±0.74^A^ | 39.4±0.75^A^ | 39.7±0.53^A^ | 13.1±0.60^A^ | 12.5±0.61^A^ | 12.8±0.44^A^ | 39.6±0.63^A^ | 39.9±0.68^A^ | 39.2±0.74^A^ | 13.4±0.51^A^ | 12.5±0.55^A^ | 12.4±0.60^A^ |
| 16:00 | 39.0±0.74^A^ | 38.9±0.76^A^ | 39.0±0.54^A^ | 13.7±0.63^A^ | 13.5±0.65^A^ | 13.5±0.46^A^ | 38.8±0.63^A^ | 39.2±0.69^A^ | 38.9±0.75^A^ | 14.2±0.53^A^ | 13.5±0.58^A^ | 13.0±0.63^A^ |
| 17:00 | 41.6±0.75^A^ | 41.2±0.76^A^ | 41.0±0.54^A^ | 12.8±0.64^A^ | 12.4±0.66^A^ | 12.8±0.47^A^ | 40.4±0.64^A^ | 41.6±0.69^A^ | 41.8±0.75^A^ | 13.8±0.55^A^ | 12.4±0.59^A^ | 11.8±0.64^B^ |
| 18:00 | 45.1±0.68^A^ | 44.2±0.70^A^ | 43.9±0.50^A^ | 10.9±0.57^A^ | 10.9±0.58^A^ | 11.3±0.42^A^ | 43.6±0.58^A^ | 44.7±0.63^A^ | 44.9±0.69^A^ | 11.9±0.49^A^ | 10.9±0.52^A^ | 10.4±0.57^B^ |
| 19:00 | 42.9±0.74^A^ | 43.6±0.75^A^ | 42.1±0.54^A^ | 12.2±0.57^A^ | 11.6±0.58^A^ | 12.4±0.41^A^ | 42.4±0.63^A^ | 43.3±0.68^A^ | 42.9±0.74^A^ | 12.9±0.48^A^ | 11.8±0.52^A^ | 11.6±0.57^A^ |
| 20:00 | 36.0±0.85^A^ | 37.3±0.83^A^ | 35.2±0.62^B^ | 16.8±0.70^A^ | 14.5±0.71^A^ | 16.2±0.51^A^ | 36.0±0.73^A^ | 37.6±0.79^A^ | 34.9±0.86^A^ | 16.3±0.59^A^ | 14.9±0.64^A^ | 16.2±0.70^A^ |
| 21:00 | 23.7±0.89^A^ | 28.7±0.91^B^ | 25.1±0.65^A^ | 24.9±0.85^A^ | 20.7±0.87^A^ | 23.7±0.62^A^ | 27.3±0.76^A^ | 26.9±0.82^A^ | 23.4±0.89^B^ | 22.5±0.73^A^ | 22.7±0.78^A^ | 24.2±0.81^A^ |
| 22:00 | 16.3±0.45^A^ | 17.9±0.46^B^ | 16.6±0.33^A^ | 30.4±0.83^A^ | 28.9±0.85^A^ | 30.2±0.60^A^ | 18.1±0.38^A^ | 16.8±0.42^A^ | 15.9±0.46^B^ | 29.4±0.71^A^ | 30.5±0.76^A^ | 29.8±0.83^A^ |
| 23:00 | 16.0±0.33^A^ | 15.9±0.33^A^ | 15.7±0.24^A^ | 30.1±0.63^A^ | 29.9±0.65^A^ | 30.3±0.46^A^ | 16.2±0.28^A^ | 15.7±0.31^A^ | 15.7±0.34^A^ | 30.5±0.54^A^ | 30.6±0.59^A^ | 29.3±0.64^A^ |
| 0:00 | 16.8±0.40^A^ | 17.5±0.41^A^ | 16.5±0.29^A^ | 28.7±0.69^A^ | 28.6±0.71^A^ | 29.6±0.50^A^ | 17.0±0.34^A^ | 16.9±0.37^A^ | 16.9±0.44^A^ | 29.1±0.59^A^ | 29.5±0.64^A^ | 28.3±0.69^A^ |

Note: This table is linked to Fig 2 and Fig 3. HFR, JE, KC represent Holstein-Friesian, Jersey, and KiwiCross breeds respectively. Lact-1, Lac-2, Lact-3 represent cows in lactation 1, 2 and 3 respectively. The means that do not share a common letter are signiﬁcantly different for the signiﬁcance level set at the p-value of 0.05.

**Table S3.** Least square means ± Standard errors of means of grazing (min/h) and rumination (min/h) for each hour over 24 hours period for different seasons.

| **Hour** | **Grazing (min/h)** | | | **Rumination (min/h)** | | |
| --- | --- | --- | --- | --- | --- | --- |
|  | **Spring** | **Summer** | **Autumn** | **Spring** | **Summer** | **Autumn** |
| 1:00 | 19.9±0.38A | 14.1±0.29^B^ | 28.5±0.43^C^ | 26.4±0.47^A^ | 32.7±0.38^B^ | 14.7±0.34^C^ |
| 2:00 | 19.2±0.37A | 14.9±0.28^B^ | 14.8±0.33^A^ | 26.5±0.48^A^ | 31.1±0.39^B^ | 27.1±0.44^C^ |
| 3:00 | 17.4±0.32A | 13.8±0.25^B^ | 13.4±0.28^C^ | 27.3±0.48^A^ | 31.3±0.39^B^ | 27.0±0.43^C^ |
| 4:00 | 14.9±0.20A | 12.8±0.14^B^ | 12.1±0.18^C^ | 28.6±0.47^A^ | 31.0±0.39^B^ | 27.8±0.43^A^ |
| 5:00 | 12.9±0.13B | 11.9±0.09^A^ | 11.8±0.11^A^ | 28.8±0.49^B^ | 30.8±0.43^A^ | 26.7±0.46^A^ |
| 6:00 | 22.8±0.20A | 12.2±0.13^B^ | 12.8±0.17^C^ | 18.4±0.49^A^ | 28.8±0.43^B^ | 24.7±0.47^C^ |
| 7:00 | 19.6±0.46A | 27.3±0.36^B^ | 29.7±0.42^C^ | 17.2±0.47^A^ | 15.3±0.43^B^ | 11.8±0.45^C^ |
| 8:00 | 32.9±0.93A | 19.7±0.88^B^ | 29.2±0.91^C^ | 9.9±0.50^A^ | 16.7±0.47^B^ | 10.6±0.49^C^ |
| 9:00 | 42.1±0.67A | 35.4±0.58^B^ | 44.8±0.64^C^ | 10.4±0.25^A^ | 9.3±0.21^B^ | 7.9±0.23^C^ |
| 10:00 | 31.8±0.72A | 41.7±0.63^B^ | 47.5±0.69^C^ | 16.1±0.43^A^ | 10.8±0.37^B^ | 8.3±0.41^C^ |
| 11:00 | 28.7±0.65A | 44.8±0.54^B^ | 38.2±0.60^C^ | 15.9±0.40^A^ | 9.3±0.33^B^ | 13.6±0.37^C^ |
| 12:00 | 39.7±0.61A | 42.0±0.45^B^ | 35.5±0.52^C^ | 12.7±0.43^A^ | 11.2±0.34^B^ | 13.7±0.37^A^ |
| 13:00 | 33.7±0.58A | 42.9±0.43^B^ | 33.7±0.49^C^ | 16.4±0.44^A^ | 10.9±0.35^B^ | 14.9±0.39^C^ |
| 14:00 | 32.8±0.55A | 41.9±0.41^B^ | 38.5±0.49^C^ | 15.7±0.39^A^ | 11.1±0.31^B^ | 13.1±0.36^C^ |
| 15:00 | 36.8±0.56A | 39.2±0.43^B^ | 42.6±0.51^C^ | 14.9±0.43^A^ | 12.4±0.34^B^ | 11.0±0.39^A^ |
| 16:00 | 38.2±0.57A | 38.1±0.43^B^ | 40.7±0.51^A^ | 14.5±0.44^A^ | 14.0±0.35^A^ | 12.2±0.40^A^ |
| 17:00 | 41.7±0.56A | 38.4±0.43^B^ | 43.7±0.51^A^ | 13.1±0.44^A^ | 14.4±0.36^B^ | 10.5±0.41^A^ |
| 18:00 | 48.2±0.54A | 39.5±0.39^B^ | 45.6±0.47^C^ | 9.1±0.41^A^ | 14.2±0.32^B^ | 9.9±0.37^C^ |
| 19:00 | 45.7±0.55B | 42.7±0.42^A^ | 40.2±0.49^A^ | 10.4±0.41^A^ | 12.9±0.33^B^ | 12.9±0.36^A^ |
| 20:00 | 33.9±0.61A | 45.0±0.49^B^ | 29.5±0.56^A^ | 16.9±0.48^A^ | 11.7±0.40^B^ | 18.8±0.44^C^ |
| 21:00 | 22.6±0.58A | 39.0±0.50^B^ | 15.9±0.55^C^ | 23.8±0.55^A^ | 15.9±0.48^B^ | 29.5±0.52^C^ |
| 22:00 | 19.3±0.37A | 18.4±0.27^B^ | 13.1±0.32^C^ | 26.6±0.54^A^ | 32.3±0.46^B^ | 30.7±0.50^C^ |
| 23:00 | 21.6±0.31B | 12.1±0.21^A^ | 13.9±0.27^A^ | 25.6±0.45^A^ | 34.6±0.37^B^ | 30.1±0.41^C^ |
| 0:00 | 23.8±0.34A | 12.6±0.24^B^ | 14.3±0.30^C^ | 24.2±0.48^A^ | 34.1±0.39^B^ | 28.6±0.44^C^ |

Note: This table is linked to Fig 4. The means that do not share a common letter are signiﬁcantly different for the signiﬁcance level set at the p-value of 0.05.

**Table S4.** Least square means ± Standard errors of means of grazing (min/h) and rumination (min/h) for each hour over 24 hours period for different supplementary feeds.

| **Hour** | **Grazing (min/h)** | | | | **Rumination (min/h)** | | | |
| --- | --- | --- | --- | --- | --- | --- | --- | --- |
|  | **Chicory** | **Pasture** | **Silage** | **Turnips** | **Chicory** | **Pasture** | **Silage** | **Turnips** |
| 1:00 | 19.2±0.24^A^ | 17.6±0.19^B^ | 15.3±0.14^A^ | 14.0±0.25^A^ | 28.4±0.24^A^ | 29.6±0.20^B^ | 28.8±0.19^C^ | 32.5±0.36^A^ |
| 2:00 | 17.9±0.22^A^ | 16.1±0.17^B^ | 15.4±0.14^C^ | 16.6±0.35^D^ | 29.2±0.23^A^ | 29.5±0.19^B^ | 27.7±0.19^C^ | 30.5±0.39^D^ |
| 3:00 | 15.6±0.18^A^ | 15.1±0.15^B^ | 13.8±0.11^A^ | 14.4±0.27^A^ | 29.8±0.22^A^ | 29.8±0.19^A^ | 27.8±0.18^B^ | 31.6±0.37^A^ |
| 4:00 | 13.8±0.14^A^ | 13.1±0.10^B^ | 12.6±0.08^A^ | 13.3±0.21^A^ | 30.3±0.21^A^ | 30.2±0.18^A^ | 28.3±0.18^B^ | 30.8±0.35^B^ |
| 5:00 | 12.4±0.09^A^ | 12.3±0.07^B^ | 11.8±0.04^B^ | 12.0±0.12^B^ | 30.2±0.20^A^ | 30.1±0.18^A^ | 28.0±0.17^A^ | 30.4±0.35^B^ |
| 6:00 | 19.6±0.21^A^ | 15.6±0.12^B^ | 14.5±0.11^C^ | 12.9±0.15^D^ | 22.0±0.22^A^ | 24.1±0.18^B^ | 24.4±0.18^C^ | 28.8±0.36^D^ |
| 7:00 | 23.2±0.26^A^ | 22.3±0.21^A^ | 26.3±0.20^B^ | 28.6±0.36^C^ | 17.3±0.19^A^ | 16.9±0.16^B^ | 13.1±0.12^C^ | 14.8±0.26^D^ |
| 8:00 | 27.3±0.29^A^ | 26.9±0.24^B^ | 27.6±0.22^B^ | 20.9±0.34^A^ | 13.0±0.15^A^ | 12.4±0.13^A^ | 12.2±0.12^B^ | 17.6±0.32^B^ |
| 9:00 | 38.4±0.33^A^ | 40.6±0.27^B^ | 43.8±0.22^C^ | 35.2±0.50^B^ | 10.7±0.16^A^ | 8.6±0.087^B^ | 8.4±0.08^C^ | 9.8±0.19^D^ |
| 10:00 | 34.9±0.35^A^ | 40.5±0.29^B^ | 43.5±0.24^A^ | 37.3±0.53^C^ | 13.9±0.21^A^ | 11.8±0.18^A^ | 10.3±0.14^B^ | 12.1±0.31^A^ |
| 11:00 | 36.9±0.34^A^ | 35.5±0.29^A^ | 38.7±0.28^B^ | 45.1±0.53^C^ | 12.6±0.19^A^ | 13.9±0.18^A^ | 12.9±0.18^B^ | 8.2±0.21^A^ |
| 12:00 | 38.1±0.33^A^ | 36.8±0.28^A^ | 37.4±0.25^A^ | 50.0±0.33^B^ | 13.3±0.21^A^ | 14.7±0.19^B^ | 12.9±0.16^C^ | 6.8±0.11^D^ |
| 13:00 | 37.5±0.34^A^ | 35.3±0.28^B^ | 35.1±0.25^C^ | 51.3±0.33^D^ | 14.3±0.22^A^ | 16.4±0.20^B^ | 14.3±0.17^A^ | 6.9±0.16^C^ |
| 14:00 | 38.9±0.32^A^ | 35.8±0.27^B^ | 38.7±0.25^B^ | 44.4±0.48^C^ | 13.3±0.21^A^ | 15.9±0.19^B^ | 12.8±0.17^A^ | 8.6±0.19^C^ |
| 15:00 | 37.9±0.31^A^ | 35.7±0.27^B^ | 41.5±0.23^C^ | 38.0±0.49^B^ | 13.9±0.21^A^ | 16.6±0.21^B^ | 11.4±0.15^C^ | 12.7±0.29^A^ |
| 16:00 | 38.8±0.30^A^ | 38.4±0.26^A^ | 40.3±0.24^B^ | 38.4±0.54^A^ | 13.9±0.21^A^ | 15.2±0.19^B^ | 12.2±0.16^C^ | 13.3±0.34^A^ |
| 17:00 | 38.9±0.31^A^ | 40.3±0.26^A^ | 42.6±0.23^B^ | 38.9±0.51^A^ | 14.5±0.22^A^ | 14.1±0.19^A^ | 11.3±0.14^B^ | 14.1±0.37^B^ |
| 18:00 | 43.8±0.29^A^ | 43.9±0.23^A^ | 44.4±0.22^A^ | 40.8±0.55^B^ | 12.1±0.21^A^ | 11.6±0.16^A^ | 10.7±0.14^B^ | 13.7±0.39^AB^ |
| 19:00 | 44.0±0.26^A^ | 46.2±0.21^B^ | 40.4±0.24^C^ | 43.8±0.50^A^ | 11.8±0.19^A^ | 10.7±0.16^B^ | 13.2±0.17^C^ | 11.8±0.35^B^ |
| 20:00 | 36.5±0.36^A^ | 41.9±0.26^B^ | 32.2±0.29^C^ | 46.8±0.41^D^ | 16.4±0.26^A^ | 13.7±0.19^B^ | 17.8±0.21^C^ | 10.1±0.29^B^ |
| 21:00 | 29.8±0.33^A^ | 30.1±0.26^B^ | 21.1±0.22^C^ | 39.4±0.52^B^ | 20.6±0.25^A^ | 21.0±0.21^A^ | 26.4±0.21^B^ | 14.9±0.39^C^ |
| 22:00 | 20.6±0.25^A^ | 18.4±0.19^B^ | 14.8±0.13^C^ | 18.0±0.32^B^ | 28.6±0.25^A^ | 30.4±0.21^B^ | 31.4±0.19^B^ | 31.8±0.36^A^ |
| 23:00 | 18.3±0.24^A^ | 16.4±0.18^B^ | 15.2±0.15^C^ | 12.4±0.15^D^ | 29.7±0.24^A^ | 30.5±0.19^B^ | 30.3±0.18^C^ | 33.5±0.31^A^ |
| 0:00 | 18.2±0.23^A^ | 17.8±0.19^B^ | 15.1±0.14^C^ | 13.8±0.25^A^ | 29.4±0.24^A^ | 29.7±0.20^B^ | 29.5±0.18^C^ | 33.4±0.35^A^ |

Note: This table is linked to Fig 5. The means that do not share a common letter are signiﬁcantly different for the signiﬁcance level set at the p-value of 0.05.
